# Elemental and Isotopic Fingerprints of Potatoes

**DOI:** 10.3390/foods14142440

**Published:** 2025-07-10

**Authors:** Cezara Voica, Ioana Feher, Romulus Puscas, Andreea Maria Iordache, Gabriela Cristea

**Affiliations:** 1 National Institute for Research and Development of Isotopic and Molecular Technologies, 67-103 Donat Street, 400293 Cluj-Napoca, Romania; cezara.voica@itim-cj.ro (C.V.); ioana.feher@itim-cj.ro (I.F.); romulus.puscas@itim-cj.ro (R.P.); 2 National Research and Development Institute of Cryogenics and Isotopic Technologies-ICSI Rm. Valcea, 4 Uzinei Str., 240050 Rm. Valcea, Romania; andreea.iordache@icsi.ro

**Keywords:** potato, elemental profile, isotopic fingerprint, geographical origin

## Abstract

Nowadays, food traceability represents an important issue in the current context of trade agreements, which influence global food prices. Many consumers prefer to pay a higher price for a traditional cultivation regime of a certain food product that comes from a certain region, appreciating the taste of the respective foodstuff. The potato is now the world’s fourth most important food crop in terms of human consumption, after wheat, maize, and rice. In this context, 100 potato samples from the Romanian market were collected. While 68 samples came from Romania, the rest of the 32 were from abroad (Hungary, France, Greece, Italy, Germany, Egypt, and Poland). The countries selected for potato sample analysis are among the main exporters of potatoes to the Romanian market. The samples were investigated by their multi-elemental and isotopic (^2^H, ^18^O and ^13^C) fingerprints, using Inductively Coupled Plasma Mass Spectrometry (ICP-MS) and Isotope Ratio Mass Spectrometry (IRMS). Then, to distinguish the geographical origin, the experimental results were statistically processed using linear discriminant analysis (LDA). The best markers that emphasize Romanian potatoes were identified to be δ^13^C_bulk_, δ^2^H_water_, and Sr.

## 1. Introduction

The potato (*Solanum tuberosum* L., family Solanaceae) is the most important non-cereal food crop globally, with more than 1 billion people worldwide consuming it [1]. The potato is now the world’s fourth most important food crop in terms of human consumption, after wheat, maize, and rice [2,3], playing a pivotal role in ensuring global food security [4]. Potatoes are gaining popularity among consumers due to their sustainability and nutritional benefits, being promoted as a healthy and versatile component of a nutritious and balanced diet [5]. It is a nutritious food, consisting of about 77% water, 16.3% starch, 0.9% sugar, 4.4% protein, 0.9% minerals, 0.59% fiber, and 0.14% crude fat, and it contains various amounts of health-promoting components such as vitamin C and phenolic compounds; it is also a good source of vitamins (B1, B3, and B6) and minerals, and it has protein content comparable to that of cereal grains [6,7]. Potatoes constitute a significant dietary source of minerals; they provide 18% of the recommended dietary allowance of K; 6% of Fe, P, and Mg; and 2% of Ca and Zn [8]. In 2017, at the European Association for Potato Research (EAPR) Conference, the following three broad concerns were identified: food security and food safety; sustainable and environmentally friendly production addressing the question of natural resource management; and innovation in practice turning scientific results into products and processes to improve the performance of agri-food systems [9]. Earth’s growing population and the increasing demand for food place unprecedented pressure on agriculture and natural resources, and feeding this population nutritiously and sustainably will require improvements to the global food system for nutritious products to consumers while minimizing today’s environmental footprint [10]. A critical challenge is how to produce more food with fewer resources, without generating negative environmental impacts. Research related to the determination of the geographical origin of foodstuffs represents an important line of study, the promulgation of international laws regulating the attribution of product origins being an indication of the growing interest in this field, especially nowadays when food traceability represents an important issue in the current context of trade agreements, which influence global food prices.

Many analytical methodologies have been employed to determine foods’ place of origin. Among them, the monitoring of trace elements content from a specific foodstuff together with the isotopic fingerprint of oxygen and hydrogen have proved to be the most effective [11,12]. In general, the research has followed diverse types of food, using different spectrometric methods, such as inductively coupled plasma mass spectrometry (ICP-MS) [13,14], high-performance liquid chromatography (HPLC) [15,16], stable isotope ratio mass spectrometry (IRMS) [17,18,19], and metabolomics based on LC-MS/GC-MS [20,21].

The determination of the geographical origin through chemical analysis coupled with sophisticated data classifying techniques is timely, and the food and nutrition database is important for an accurate evaluation of nutrient intake from dietary surveys. Potato production depends on the intensive use of fertilizers and pesticides, leading to serious environmental and food safety concerns [22]. The content of micronutrients and trace elements of potato tubers is influenced by factors such as the type of production system used for their growth, the genotype of the plants, and the weather conditions during the growing season. Cross-contamination of the human food chain with risk elements such as heavy metals is an important problem, as these elements that are mainly anthropogenic in nature not only affect the nutritional value of food, but they also have harmful effects on the health of consumers [23]. An increasing number of scientific studies are aimed at developing analytical/chemometric strategies for the geographical classification of potatoes [4,24,25,26].

The economic crisis has accelerated the prices of the main food commodities. Nevertheless, many consumers prefer to pay a higher price for a traditional cultivation regime of a certain product (potatoes, in this case) that comes from a certain region, appreciating the taste of the respective foodstuff. For the better promotion of Romanian products, there is a need to develop new fast and precise analytical approaches that are capable of differentiating the quality and origin of Romanian potatoes. In this context, the aim of this study was to characterize the potatoes from the Romanian market, according to their geographical origin, by determining the mineral and trace element concentrations, along with stable isotope signatures from these samples. Then, the entire experimental data set was processed using a chemometric supervised method, linear discriminant analysis (LDA), to emphasize the most important markers that could distinguish the geographical origin of potatoes.

## 2. Materials and Methods

### 2.1. Sampling

A total of 100 potato samples were investigated using the mass spectrometry technique, determining their elemental and isotopic profiles. Samples were collected from both supermarkets and family farms (rural regions). Of these, 68 samples originated from Romania (39 were white-skinned potatoes and 29 red-skinned), and the rest of the 32 were from abroad (Hungary, *n* = 6; Greece, *n* = 7; France, *n* = 8; Italy, *n* = 2; Germany, *n* = 2; Egypt, *n* = 2; Poland, *n* = 3; Tunisia, *n* = 1; Netherlands, *n* = 1). Among the imported samples, 23 were white-skinned (Hungary, *n* = 2; Greece, *n* = 6; France, *n* = 6; Italy, *n* = 2; Germany, *n* = 2; Egypt, *n* = 2; Poland, *n* = 2; Tunisia, *n* = 1), and nine samples were red-skinned (Hungary, *n* = 4; Greece, *n* = 1; France, *n* = 2; Poland, *n* = 1; Netherlands, *n* = 1). The countries selected for potato sample analysis are among the main exporters of potatoes to the Romanian market. These countries were chosen based on their significant presence in Romania’s potato imports, making them relevant for assessing the quality and characteristics of imported potatoes. All samples were stored in clean polyethylene bags according to their type and brought to the laboratory for analysis.

**Figure 1:**
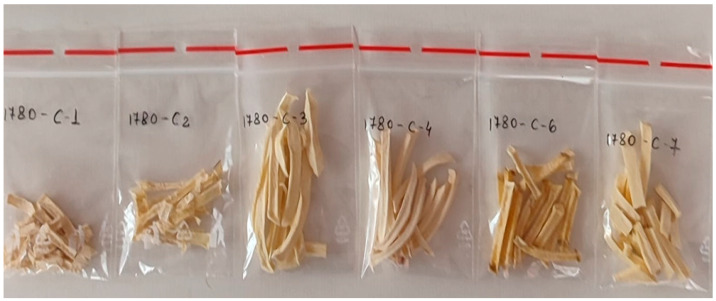
Potato sample after the step of water extraction.

Before determining the elemental and isotopic compositions of the samples, in the laboratory, from each potato sample, the water was extracted by a procedure that used cryogenic distillation under vacuum [11]. At the end of this procedure, the obtained samples were completely dry, without any water content (Figure 1).

Then, from the extracted water, ^2^H_water_ and ^18^O_water_ signatures were determined. The dried part of each potato sample was divided into two lots, one for subsequent elemental concentration determination and the other one for ^13^C_bulk_ isotopic investigation. Both parts were prepared separately, according to proper protocol.

### 2.2. Elemental Measurements

For ICP-MS, ultrapure deionized water (resistivity of 18 MΩ cm^−1^) from a Milli-Q analytical reagent grade water purification system (Millipore, Bedford, MA, USA) and ultrapure nitric acid (HNO_3_, Merck, KGaA, Darmstadt, Germany) 60% were used. A total of 0.1 g (accurate to 0.0001 g) of potato sample was placed into a digester and mixed with 3 mL of concentrated nitric acid with the controlled program (pressure and temperature) for 12 min at 200 °C, using a microwave oven model Speedwave (Berghof Products and Instruments Ltd., Eningen, Germany). After digestion, each digest was transferred with ultrapure water to 50 mL. The digested solution was analyzed using an inductively coupled plasma mass spectrometer.

For the characterization of the studied food samples, an ELAN DRC (e) inductively coupled plasma mass spectrometer was used, produced by Perkin Elmer (Pure Plus, Billerica, MA, USA), with a Meinhard nebulizer and glass cyclonic spray chamber for pneumatic nebulization. Primary analysis was performed using the semi-quantitative method Total Quant, a method that is recommended in fingerprint analyses where large amounts of analytes are essential. Semi-quantitative analyses are a particularly useful tool for the rapid analysis of the entire mass spectrum, especially for a sample with an unknown composition. The rare earth elements (REEs) were determined using the Total Quant method.

The precise determination of heavy metals in foods is very important because there is a narrow margin of safety between adequate intake and overconsumption. Several parameters were evaluated to validate the analytical method for the quantitative determination of the major and trace elements in food samples. Linearity was established using calibration curves that were obtained for aqueous reference solutions for all the analytes, and the linearity values of the six points were found to be acceptable (R > 0.999). The limits of detection (LOD) and quantification (LOQ) were experimentally calculated as three and ten times the standard deviation (SD) of the blank determination, divided by the slope of the analytical curve. The method was previously validated, and the LOD for each target metal was measured in mg/kg as follows: 0.5 (Na), 1.0 (Ca, Mg), 5.0 (K), 0.001 (Al, Co, Sr, Ba), 0.003 (Fe), 0.005 (As, Cd, Pb, Ni, V), 0.006 (Cr, Cu, Mn, Zn). The accuracy (as recovery) and precision (as relative standard deviation [RSD]) of the procedure were determined by analyzing two certified reference materials, CRMNCS ZC85006 tomato trace elements and CRM IAEA-359 cabbage trace elements (China National Analysis Center for Iron and Steel, Beijing, China), which were mineralized similarly to the samples. The results showed that the data obtained by ICP-MS were in agreement at the 95% confidence limit. The recovery ranged from 80 to 110%, which testified the applicability of the method to potatoes. The mean relative standard deviations of replicate samples were under 10%. The agreement between the certified and measured values was good, demonstrating the satisfactory performance of the developed method.

The performance of the ICP-MS analysis strongly depends on the operating conditions. The ELAN DRC (e) uses chemical resolution to eliminate plasma-based polyatomic species before they reach the quadrupole mass spectrometer. This ion molecule chemistry uses a reaction gas to “chemically scrub” polyatomic or isobaric species from the ion beam before they enter the analyzer, resulting in improved detection limits for traditional difficult elements, including As, Cr, Fe, Se, and others. Cu, Cr, and Fe were determined using the DRC mode with methane at 0.8 L/min. in the reaction chamber, and a rejection parameter value of 0.7 was applied.

The operating conditions were as follows: nebulizer gas flow rate = 0.92 L/min; auxiliary gas flow = 1.2 L/min; plasma gas flow = 15 L/min; lens voltage = 7.25 V; ICP radio frequency power = 1100 W; CeO/Ce ratio = 0.028; and Ba^++^/Ba^+^ = 0.029. The instrument was tuned daily with an Elan 6100 Setup/Stab/Masscal Solution (a solution of 10 µg/L Mg, Cu, Cd, In, Ba, Ce, Pb, and U in 1% HNO_3_) following the manufacturer’s specifications.

### 2.3. Stable Isotope Measurements

Each dried potato sample was crushed, using a ball mill, to obtain a fine powder. Then, 5 mg samples were converted to CO_2_ by dry combustion, in oxygen excess for 3 h, at 550 °C. The resulting CO_2_ was purified from other combustion gases by cryogenic separation and subsequently measured by the isotope ratio mass spectrometry (IRMS) technique.

The isotopic composition (signature or fingerprint) is denoted in delta values δ versus international standards, following Equation (1) [27]:

(1) $$\delta^{i}X=\left(\frac{R_{sample}}{R_{standard}}-1\right)1000$$

where *i* represents the mass number of the heavier isotope of the element X (^2^H, ^18^O, ^13^C), *R_sample_* is the isotope number ratio of a sample (^2^H/^1^H; ^18^O/^16^O; ^13^C/^12^C), and *R_standard_* is that of the international standard (Vienna Standard Mean Ocean Water, V-SMOW, for δ^2^H and δ^18^O, and Vienna Pee Dee Belemnite, V-PDB, for δ^13^C). The delta values are multiplied by 1000 and are expressed commonly in units “per mil” (‰) or, according to the International System of Units (SI), in units milli Urey (mUr) [28].

To determine the δ^13^C isotopic signature of CO_2_ derived from potatoes, an isotope ratio mass spectrometer (Delta V Advantage, Thermo Scientific, Waltham, MA, USA) equipped with a dual inlet system was used. Prior to daily sample analysis, a working standard was measured and calibrated against the NBS-22 oil certified reference material (δ^13^C_VPDB_ = −30.03‰) provided by the IAEA Vienna (International Atomic Energy Agency). The measurement of each sample consisted of two acquisitions, each of them containing eight cycles of analysis. The standard deviation per analysis was consistently below 0.05‰, and the associated measurement uncertainty was ±0.3‰.

A liquid–water isotope analyzer (DLT-100, Los Gatos Research, San Jose, CA, USA) was used for measurements of ^2^H and ^18^O from potato-extracted water. For ^13^C/^12^C determinations, the analyses were performed using an isotopic ratio mass spectrometer (IRMS, Delta V Advantage, Thermo Fisher Scientific, Waltham, MA, USA). A set of five reference materials (RM), calibrated against the Vienna Standard Mean Ocean Water international standard, V-SMOW, was used for calibration purposes, covering the following broad isotopic range: RM1, δ^18^O = −19.57 ± 0.1‰ and δ^2^H = −154.1 ± 1‰; RM2, δ^18^O = −15.55 ± 0.1‰ and δ^2^H = −117.0 ± 1‰; RM3, δ^18^O = −11.54 ± 0.1‰ and δ^2^H = −79.0 ± 1‰; RM4, δ^18^O = −7.14 ± 0.1‰ and δ^2^H = −43.6 ± 1‰; and RM5, δ^18^O = −2.96 ± 0.1‰ and δ^2^H = −9.8 ± 1‰. All samples were measured in duplicate. Each analysis consisted of 7 acquisition cycles; the first 3 cycles were discarded to eliminate potential memory effects. The final result was calculated as the average of the remaining 4 cycles. The maximum standard deviation of the isotopic analysis was ±0.2‰ for δ^18^O and ±1.0‰ for δ^2^H, respectively.

### 2.4. Statistical Analysis

Chemometric experimental data processing was performed using SPSS Statistics version 24 (IBM, New York, NY, USA) software. One of the most widely used supervised methods for fingerprinting purposes is linear discriminant analysis (LDA). The aim of the method is to find the samples that have the most similar characteristics (low distance) and that are different from the rest (longer distance).

Alongside the experimental data set, a new variable is created for each category that is under study. Within this new variable, each sample receives a code specific to its characteristics. The results of this method are discriminant functions (DFs), which are linear combinations of each representative variable. This chemometric model is validated using “leave-one-out cross validation”, which means that each sample is tested as a new one. The results of initial and cross-validation steps are expressed in percentages; the higher the values are, the more robust the model is. In this study, LDA was applied for geographical classification and for highlighting the differences between red and white potatoes.

## 3. Results and Discussion

### 3.1. Elemental Results

The quantitative contribution of macro elements represented more than 80% of all investigated minerals. Considering the major elements, it can be observed that potato is an important source of potassium (K), which is the most abundant element compared with calcium (Ca), magnesium (Mg), sodium (Na), and phosphorous (P). This observation is in agreement with other studies regarding the macro element profile of potatoes (Table 1), with potassium variability depending on the geographical origin of the potato. The decrease in mean concentrations was in the following order (g/kg, dry weight): K > P > Mg > Ca > Na. The differences in mineral nutrient compositions between potatoes could be attributed to the adaptation mechanisms of potato accession to the growing conditions [29].

Potassium plays a key role in good overall plant health and is essential for the completion of various physiological and metabolic functions in plants; it is considered the second most abundant nutrient after nitrogen, and it is even more abundant than phosphorous [36]. Potato crops require a high amount of potassium to achieve the ideal yield and quality, with the role of potassium in potato plant growth, in the biochemistry of starch synthesis, and in tuber quantity and quality being known [37]. It is the nutrient taken up in the largest quantity by potatoes throughout the growing season, influencing the transport of nutrients and the movement of carbohydrates from the leaf to the tuber [38,39]. Some factors such as the environment as well as crop management can affect the tuber potassium content. For example, drought conditions were found to increase K by about 12% [40]. Potassium levels in the analyzed potatoes (5.994–23.412 g/kg) were in line with those given by other studies [8,30,33,34,35].

Phosphorous plays a fundamental role in crop and vegetable physiology, the most important being the energy storage and transfer of it in plants [41,42]. The potato crop demands high phosphorus levels, and these are correlated with increased root development; thus, when phosphoric acid increases in the soil, plant roots can spread extensively [43,44]. The management of fertilizer phosphorus is a critical component of potato production systems, so the total phosphorous concentration in potato changes based on the growth stage and on the fertilization and applications of manure with high P concentrations [41,45]. The soil is a very important factor, with the sorption and desorption properties of this governing the phosphorous uptake by potatoes. There are studies that showed that the phosphorous solubility increased as soil pH changed from acid to neutral pH [42,46], that the potato production would be tolerant of acid soil as long as the aluminum concentration is low, or that the acidic soils rich in aluminum and iron ions would mainly fix and precipitate P as an Al–Fe–P component [41,42]. The total content of P in most plant tissues ranges from 0.1 to 1% [47]. The mean content of studied potatoes (1.707 g/kg) is within the range of contents reported by other authors [8,30,31,32,33,34,35].

Calcium is an essential macronutrient for plant growth and development, with vital structural, metabolic, and signaling roles [48]. Potatoes were found to take up significantly different quantities of calcium depending on the variety of potato studied; thus, in the studied potatoes, the range was 0.087–3.131 g/kg, with a mean of 0.453 g/kg, in line with some papers [8,30,31,32,33,34,35].

Magnesium is an essential nutrient for potato plants, playing a crucial role in various metabolic processes. It is a critical nutrient for potato production, affecting many aspects of plant growth and development, with a vital role in chlorophyll synthesis and the enzyme activation involved in energy metabolism and protein synthesis [49]. Our results emphasized that the Mg concentration (g/kg) ranged between 0.475 and 5.400 (mean of 1.109). The literature indicates similar values [31,33]. It seems that the content of Mg is connected to the amount of rainfall, irrigation and fertilization protocols, average climate and temperature, sun, light, and irradiation [50].

Trace elements are indispensable for the cultivation of quality plants and are essential for their good growth. Even if they are found in small quantities, they form a balance corresponding to their growth, with a role in plant metabolism [51]. The concentrations of some of the trace elements in the studied potatoes were in the following ranges (mg/kg): <0.001–0.515 ± 0.036 (Li); <0.001–0.023 ± 0.002 (Be); <0.001–0.661 ± 0.035 (Co); 0.019 ± 0.001–0.585 ± 0.033 (V); <0.001–11.236 ± 0.995 (Ti). The mean concentration for boron and molybdenum was 6.206 ± 0.455 mg/kg and 0.384 ± 0.022 mg/kg. Boron has a key role in the growth process, but optimizing the utilization of calcium and molybdenum can be important at low soil pH levels. In the case of an insufficient supply of molybdenum, the chlorophyll content of plants decreases, and the process of photosynthesis becomes difficult. Comparative reported values for the investigated microelements in studied potatoes (this study versus other references) are presented in Table 2. Generally, potatoes contain moderate amounts of iron (Fe), which should be readily bioavailable because potatoes have very low phytic acid concentrations, unlike some other crops. Vitamin C from potatoes is essential for the absorption of iron in the body (potatoes contain 45% of the recommended daily intake of vitamin C) [50]. The concentrations of Fe in studied samples (mean of 73.659 mg/kg) were in line with [34,52,53] or even higher [33] than those from other studies. The elements zinc (Zn), cooper (Cu), chromium (Cr), and nickel (Ni), from the trace elements category were recorded in investigated samples, and the accumulation pattern in studied potatoes was in the order Zn > Cu > Cr > Ni. The same order was found in other studies [54]. The mean concentration of these elements (9.344 mg Zn/kg, 4.580 mg Cu/kg, 3.80 mg Cr/kg, 2.961 mg Ni/kg) was in line with the literature [31,55,56]. The highest concentrations of Cr and Cu in potatoes were 21.455 mg/kg and 55.544 mg/kg. The greatest accumulation of Cr in potatoes could be caused by the influence of soil salinity on the mobility of heavy metals or by the fact that it could be contaminated by Cr in the irrigation water, even when grown in unpolluted soil. Regarding Cu, it also plays a role in metabolism and protein synthesis, but the excess of this in the soil induces stress in the plants, delays their growth, and causes chlorosis of the leaves. Manganese, like zinc, is important from the point of view of cultivation, as it participates as an activator in the metabolic process of plants, and zinc has an important role in nitrogen assimilation and metabolism, contributing to the formation of starch. Mn concentration tends to vary with environmental conditions, and toxicity due to excess Mn can damage potato plant growth, especially when the pH level is low [57,58]. Bedoya-Perales et al. (2023) [59] reported similar levels of Mn (1.086–15.145 mg/kg), Ba (0.070–1.350 mg/kg), and Cu (0.970–1.534 mg/kg).

The potential of information obtained from rubidium content in potato culture is still unexploited. This element plays a crucial role in various physiological processes of potato plants, including nutrient uptake and assimilation; it contributes to the accumulation of starch, vitamins, and minerals, enhancing the nutritional value of potatoes. Rubidium has been found to enhance potato plants’ tolerance to abiotic stresses, such as drought and salinity [60]. Strontium can be efficiently accumulated by plants, with its content being strongly positively correlated with its concentration in the growth medium; thus, the strontium in the nutrient medium can help to functionalize some plants [61,62,63]. The wide range of strontium concentrations obtained in the studied potato samples (0.308 ± 0.020–14.586 ± 1.120 mg/kg) highlights the fact that the process of nutrient uptake from a medium supplemented with strontium is complex and based on a lot of factors (e.g., plant species, time, balance between nutrients in the growth medium) [64,65]. Strontium can be used as a tracer for calcium uptake and allocation in the short-term, making it a powerful tool for studying factors that govern calcium allocation to plant organs [66].

Monitoring the content of toxic and potentially toxic elements is one of the most important aspects of food quality assurance [67]. The vegetable uptake of metals is one of the major pathways through which soil metals enter the food chain. The accumulation of toxic metals in soils is a major factor determining the high levels of these metals found in crops, and potatoes are in close contact with the soil and stay in the soil for several months before being cooked [54]. The mean concentrations of toxic metals in studied potatoes were (mg/kg) 0.028 (As), 0.09 (Cd), 0.026 (Hg), and 0.084 (Pb), respectively. Similar results were also reported for Czech potatoes (mg/kg) 0.5–0.7 (Pb), 0.08–0.3 (Cd) [53], or 0.06 (As), 0.6 (Pb), and 0.04 (Cd) [55]. Ref [52] reported similar levels (mg/kg,) of As (0.037–0.184), Pb (0.069–0.861), and Cd (0.021–0.076). The ability of the potato to absorb toxic metals from the soil was small; thus, the concentration of these elements in potatoes was below the maximum threshold of 0.1 mg/kg Pb and Cd [68,69].

Rare earth elements (REEs) are non-essential elements for living systems, but the long-term consumption of food contaminated with REEs may cause chronic poisoning. The lanthanoids, together with yttrium and scandium, form the group of REEs. REEs from agriculture and the natural environment could be ingested by humans throughout the food chain; thus, it is necessary to investigate the levels of REEs in food items. The REE mean concentrations in studied potatoes were as follows (mg/kg): 0.025 (Sc); 0.008 (Y); 0.018 (La); 0.040 (Ce); 0.004 (Pr); 0.015 (Nd); 0.002 (Sm); 0.0006 (Eu); 0.053 (Gd); 0.0002 (Tb); 0.001 (Dy); 0.0003 (Ho, Er); 0.0006 (Tm); 0.001 (Yb); and 0.0001 (Lu).

The comparative study of the elemental profile of autochthonous vs. imported potatoes presents the fact that the local potatoes had higher concentrations of some elements (Mg, Al, Ti, V, Cr, Fe, Ni, Rb, Sr, Ba, toxic metals) and lower concentrations of other elements (Na, P, K, Ca, Mn, Co, Cu, Zn) than those imported.

Regarding the potatoes’ skin color, the mean concentration of most elements was highest in white-skinned potatoes compared to the reds. Ref. [70] reported similar levels of K, P, Ca, and Mg (g/kg) (21.49; 3.07; 0.33; 1.17) and Zn, Cu, and Mn (mg/kg) (24.49; 7.56; 6.84) for the white potatoes. The mean concentrations of iron and zinc in red potatoes were 56.47 and 8.47 mg/kg, in line with the literature (19.28–63.94 mg/kg and 7.03–39.2 mg/kg) [71]. Additionally, there were some differences between different types of potatoes: (i) the average concentrations of macro elements (Na, P, K, Ca) or Mn, Cu, and Zn were higher in foreign white potatoes than in Romanian white potatoes; (ii) the average concentrations of most elements (V, Cr, Mn, Fe, Co, Ni, Cu, Zn, etc.) were lower in foreign red potatoes than in Romanian red potatoes.

### 3.2. Stable Isotopes Results

**Figure 2:**
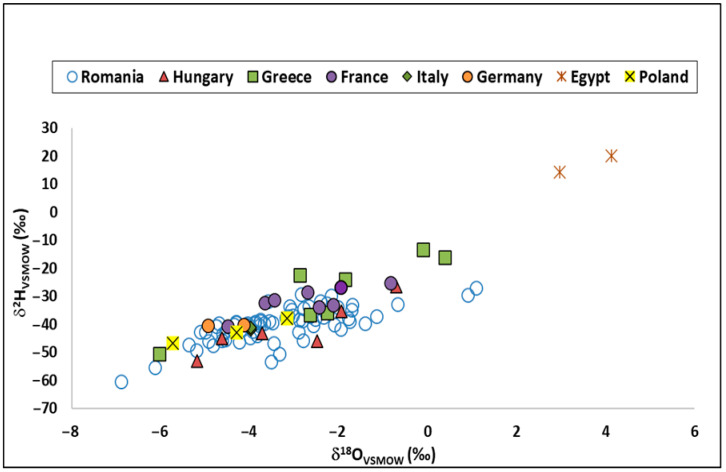
Isotopic fingerprint (δ^2^H versus δ^18^O) for studied potatoes.

To obtain qualitative data linked to potatoes’ geographical origin, isotopic compositions of ^2^H_water_ and ^18^O_water_ are plotted in Figure 2. An important difference was observed between the ^2^H_water_ and ^18^O_water_ isotopic fingerprints of the Romanian and foreign potato samples.

For Romanian samples, δ^2^H_water_ ranged from −62.4 to −32.9‰ (mean value of −39.9‰), and δ^18^O_water_ ranged between −6.9 and −1.7‰ (mean value of −3.3‰). These values are in the range of those published by [72] for Romanian conventional potatoes. Additionally, ref. [26] recorded similar results of δ^18^O for potato samples coming from two Slovenian macro-regions (Alpine and Dinaric). Regarding samples from abroad, the isotopic signature of ^2^H had a range of variation from −53.1 to +20.1‰ (mean of −31.6‰), and that of ^18^O was from −6.0 to 4.1‰ (mean of −2.7‰).

The isotopic compositions of ^2^H and ^18^O in water vary predictably with geographical origin, decreasing from low-latitude, low-elevation coastal regions to inland, high-latitude, mountainous regions [12,73]. The ^18^O and ^2^H content of potato water reflects the ^18^O and ^2^H fingerprint of the groundwater at the location where the plants are grown. Thus, the δ^18^O and δ^2^H measurements of potato water should provide valuable information about the sample’s origin.

The sample with the highest isotopic values of hydrogen (average of 17.2‰) and oxygen (average of 3.5‰) is a conventional potato, labeled “Egypt”. This is not surprising, as Egypt has a dry climate that is hot and dominated by desert and a low latitude (30°06′ N) compared to the other sample countries of origin. Higher evapotranspiration rates from this dry region can lead to enriched values of ^2^H and ^18^O [74]. Many potato samples were imported from different countries for the Romanian market, and this fact is also sustained by the isotopic results for these vegetables. Elevated values for δ^2^H_water_ and δ^18^O_water_ were also obtained for samples originating from Greece or France, depending on the climatic particularities of the production area. Four samples from Greece and a few from France fit with Romanian potato isotopic values. This overlap could be attributed to the limited number of samples from each country and the lack of precise information about the cultivation locations, rather than a mislabeling of origin. Therefore, while the possibility of a false declaration cannot be entirely excluded, the observed similarity in isotopic signatures is more likely due to insufficient exact field information and direct traceability (such as sourcing the potatoes directly from farmers) and shared environmental characteristics. Regarding the Romanian potatoes, the lowest value was recorded for a sample produced in the Eastern Transylvanian region (Harghita), where the climate is colder and altitude is higher (1300–1400 m above sea level). Samples from Hungary overlap with those from Romania, with these two countries being neighbours, and the agricultural areas from where the potato samples came from are probably not far away from each other. This explanation could be sustained by the published paper [75], showing that a more important geographic differentiation of food samples using isotope and elemental compositions can be obtained by comparing products originating from areas far away from each other, whereas correct attribution is usually limited if production geographical regions are nearby locations. Additionally, a similar case was recorded for the isotopic signature of pork meat samples that came from the western part of Romania, near the Hungarian border, which presented similar results to those from Hungary [76].

The percentage of water in the potato samples varied between 72.4% and 93.5% (with an average of 80.9%) for the samples from Romania and between 75.0% and 90.6% (the average being 82.3%) for those from abroad.

**Figure 3:**
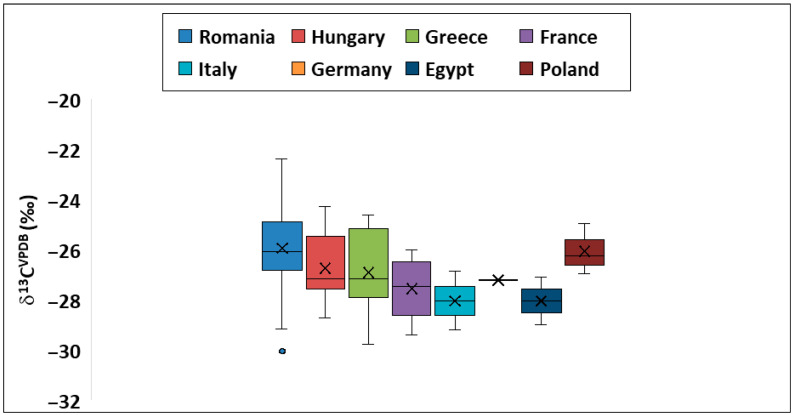
Isotopic fingerprint (δ^13^C_bulk_) for studied potatoes. The circle represents the outliers and the cross symbol is the mean of data.

δ^13^C_bulk_ values for the investigated samples are presented in Figure 3.

The isotopic signature of ^13^C in terrestrial plants varies depending on their photosynthetic pathway. There are two primary types of photosynthesis, C3 (Calvin cycle) and C4 (Hatch-Slack cycle), which are distinguished by the enzyme involved in the initial carboxylation step and the number of carbon atoms in the first stable compound formed (three for C3 plants and four for C4 plants) [12]. The C3 pathway is found in most plant species on our planet (vegetables, fruits, and the majority of cereals) and exhibits ^13^C values ranging from −30 to −23‰. In contrast, C4 plants such as maize, sugarcane, millet, and sorghum have higher ^13^C isotopic signatures, typically between −14‰ and −12‰.

δ^13^C_bulk_ values of Romanian samples ranged from −30.0 to −23.3‰ (mean of −25.9‰), and this variation interval was consistent with that of C3 plants [26]. While the mean values of samples from Hungary (−26.7‰), Greece (−26.9‰), France (−27.5‰), Italy (−28.0‰), Poland (−26.0‰), Germany (−27.2‰), and Egypt (−28.0‰) were numerically lower (more negative) than that of the Romanian samples, the standard deviations of these groups overlap. It can be observed that there is a general trend of slightly less negative δ^13^C values in Romanian samples than those of the other countries, as the differences were not statistically significant due to the large overlap in variability. A Kruskal–Wallis test was conducted in order to detect differences among different countries. The p value was 0.001. Even if the ^13^C isotopic compositions are strongly related to the photosynthetic pathway, the differences in the ^13^C_bulk_ isotopic fingerprint of potato samples across countries could also be assigned to variations in local growing conditions, such as precipitation quantity, the mean annual temperature, relative humidity, and nutrient availability [77]. Opatic et al. [26] reported a mean value of −25.0‰ for potato samples from a macro-region (Alpine) in Slovenia, a value that is similar to that obtained for Romanian investigated samples (−25.9‰). In addition to environmental factors (precipitation, temperature, and relative humidity), the varietal characteristics of potatoes may also influence δ^13^C values. Previous studies on various agricultural products (e.g., pistachios [78], apples [79], and hazelnuts [80]) have demonstrated significant isotopic variation attributable to cultivar differences. In our study, the analyzed potato samples included both red- and white-skinned varieties, and this varietal diversity could contribute to the observed isotopic differences across countries.

### 3.3. Chemometric Results

For statistical interpretation, some of the analyzed elements were removed from the initial data set, such as Be, Sc, Ge, Zr, Pd, Ag, Te, Cs, Sm, Eu, Gd, Tb, Dy, Ho, Er, Tm, Yb, Lu, Hf, Ta, W, Re, Os, Ir, Bi, Th, and U. For the LDA method, Wilks Lambda was chosen for measuring the similarity among analyzed potatoes samples. Thus, for geographical classification, each sample received one of the following codes corresponding to its origin: code 1 for Romanian samples or code 2 for foreign samples.

The percentage obtained for each initial classification was 89.0%, while for cross-validation it was 82.0%. Significant markers were δ^13^C_bulk_, δ^2^H_water_, and Sr. When LDA is computed, some linear discriminant functions are compiled, and significant markers (selected among measured characteristics) have different coefficients. In our case, the selected markers δ^13^C, δ^2^H, and Sr had coefficients of 0.422, 1.001, and 0.843, respectively.

**Figure 4:**
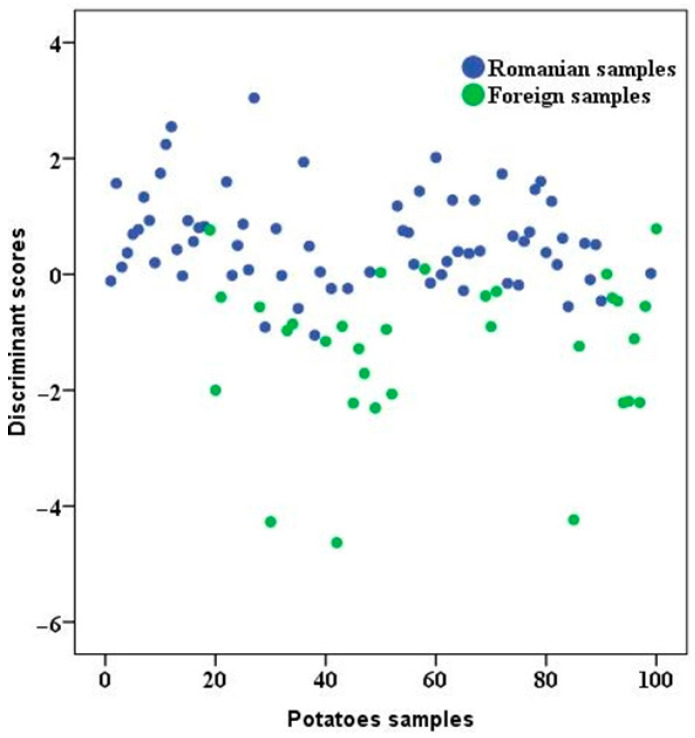
Geographical classification of potato samples (Romanian and foreign).

From the classification results, it could be observed that the five samples of Romanian origin were placed into the foreign group, and six foreign samples were put in the Romanian group. The graphical representation of potato samples according to origin is presented in Figure 4.

**Figure 5:**
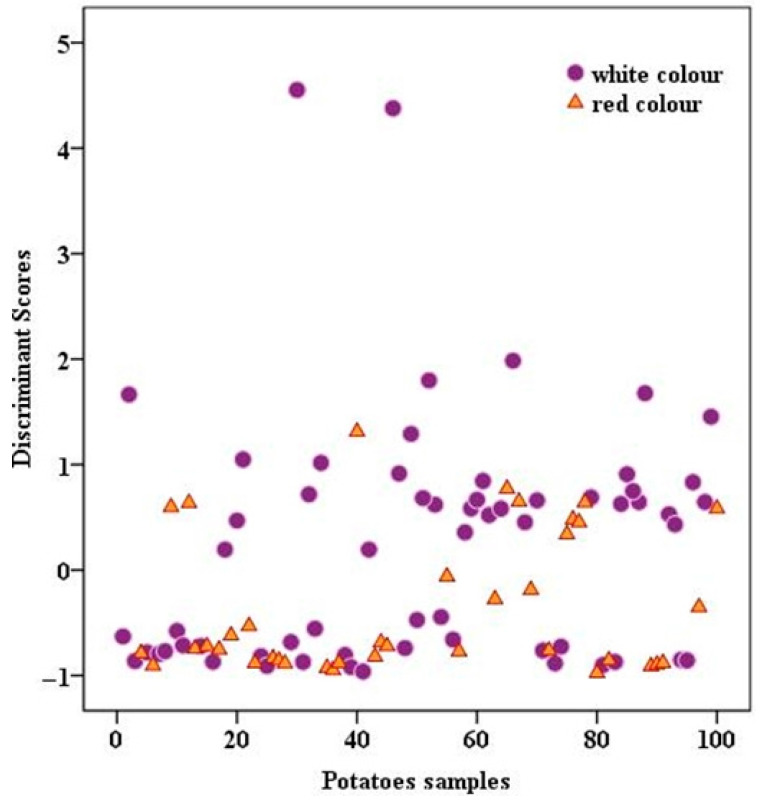
Classification of potato function by their skin color (white and red).

The classification of potato function by their skin color (white and red) is presented in Figure 5. This association between ICP-MS and IRMS might not be the most suitable instrument for fulfilling the purpose of differentiating between skin colors. The analyzed potatoes presented similar isotopic and multi-element contents, and significant differences could not be observed. Future studies might imply other analytical techniques capable of better classifying the potatoes according to skin color.

## 4. Conclusions

In this study, 100 potato samples were investigated from the elemental and isotopic point of view, using inductively coupled plasma mass spectrometry and isotope ratio mass spectrometry. The quantitative contribution of macro-elements represented more than 80% of all minerals. The decrease in concentrations of the potato sample was in the order of K > P > Mg > Ca > Na. The content of toxic metals (As, Cd, Pb, Hg) in the analyzed foodstuffs was low, and they can be safely consumed. The isotopic results of these vegetables support the mention on the label that many potato samples were imported from different countries for the Romanian market. Chemometrics were used to highlight the most important features able to predict the geographical origin of potatoes. The best predictors for geographical differentiation of potato samples were δ^13^C_bulk_, δ^2^H_water_, and Sr. Unfortunately, the differentiation according to potato skin color could not be performed using available analytical parameters, and this represents a good challenge for future studies in this direction. The results of this study may provide useful baseline data on the variations of some elements in Romanian and commercially available imported vegetables and may initiate further studies on the nutritional and toxicological effects of the commercially available foodstuffs.

## Figures and Tables

**Table 1:** Comparative reported values for the investigated macro-elements in studied potatoes: this study versus other references.

References	U.M.	Elements				
		Na	Mg	P	K	Ca
This study (range, mean)	g/kg	0.004–1.096 (0.087)	0.475–5.4 (1.109)	0.612–4.161 (1.707)	5.994–23.412 (13.742)	0.087–3.131 (0.453)
[30]	g/kg	0.141–0.333	1.134–1.453	-	21.943–28.22	0.341–0.712
[8]	g/kg	0.102–0.468 (0.276)	0.81–1.44 (1.14)	2.96–4.79 (3.69)	20.39–32.46 (25.09)	0.19–0.79 (0.44)
[31]	g/kg	-	0.38–8.00	3.15–4.95	12.95-26.60	0.02-1.00
[32]	g/kg	-	-	-	-	0.271–1.092
[33]	g/kg	0.007-0.12	0.95–1.07	1.62–2.20	19.63–24.66	0.10–0.15
[34]	g/kg	-	0.4–2.9	1.3–3.9	4.8–20.1	0.20–1.0
[35]	g/kg	-	0.8–2.2	2.4–5.2	15.0–26.9	0.1–0.7

**Table 2:** Comparative reported values for the investigated microelements in studied potatoes: this study versus other references.

References	U.M.	Elements					
		Fe	Zn	Cu	Cr	Ni	Mn
This study (range, mean)	mg/kg	16.981–255.792 (73.659)	1.779–33.642 (9.344)	<0.001–55.544 (4.580)	<0.001–21.455 (3.80)	<0.001–13.610 (2.961	2.412–54.627 (7.467)
[31]	mg/kg	118.5–615.5	17.4–78.1	1.1–13.5	-	-	11.1–83.7
[54]	mg/kg	208.9–380.5	34.4–54.0	8.9–13.5	2.8–7.8	16.8–45.5	16.8–45.5
[55]	mg/kg	-	16.1	4.3	0.19	0.58	-
[56]	mg/kg	-	1.4	2.52	0.39	0.25	-
[59]	mg/kg	-	-	0.970–1.534	-	-	1.086–15.145
[53]	mg/kg	48.87–72.64	13.80–18.88	3.07–5.43	-	2.02–3.55	7.20–13.06
[52]	mg/kg	5.25–200.67 (94.531)	13.03–26.41 (18.251)	-	**-**	-	**-**
[33]	mg/kg	18.0–24.0	13.0–15.0	2.0–3.0	-	-	6.0–8.0
[34]	mg/kg	23.60–76.90	6.80–36.0	3.80–23.10	-	-	7.20–28.40
[35]	mg/kg	12.2–43.6	5.9–26.9	2.6–10.8	-	-	3.9–11.7

## Data Availability

The original contributions presented in this study are included in the article. Further inquiries can be directed to the corresponding author.

## Abbreviations

The following abbreviations are used in this manuscript:

ICP-MS	Inductively coupled plasma mass spectrometry
IRMS	Isotope ratio mass spectrometry
LDA	Linear discriminant analysis
